# Supplementary material for: A novel ABC fractional-order mathematical model for malaria transmission dynamics incorporating treatment-seeking behavior
Source: PLoS One. 2025 Jun 18;20(6):e0319166. doi: 10.1371/journal.pone.0319166 (PMC12176305; doi:10.1371/journal.pone.0319166)
Supplement: S1 Data — (DOCX) [file pone.0319166.s001.docx]

**Parameters Description of the Model with their values**

In our study, we utilized secondary data obtained from previously published literature. We carefully collected and compiled this data, which includes various parameters and values relevant to malaria transmission dynamics. The data, along with their corresponding references, are summarized in the following table. This approach allowed us to leverage established findings from the literature to enhance the robustness and reliability of our mathematical modeling and numerical simulations. The references cited in the table provide the original sources of the data, ensuring transparency and accuracy in our research.

**Table 2.** **Parameters description of the model with their values**

| ***Parameters*** | ***Descriptions*** | ***Units*** | ***Values*** | ***References*** |
| --- | --- | --- | --- | --- |
| $\Lambda_{h}$ | Recruitment rate for humans | $/month$ | 0.000215 | $[39]$ |
| $\Lambda_{m}$ | Recruitment rate for mosquitoes | $/month$ | 0.07 | $[39]$ |
| $\mu$ | Natural death rate of humans | $/month$ | 0.000045 | $[40]$ |
| $\eta$ | Natural death rate of mosquitoes | $/month$ | 0.0477 | $[40]$ |
| $q$ | Average per capita biting rate of mosquitoes | $/month$ | 0.33 | $[40]$ |
| $\alpha_{1m}$ | Probability of transmission of infection from $I_{h}$ to $S_{m}$ | - | 0.8333 | $[41]$ |
| $\alpha_{2m}$ | Probability of transmission of infection from $T_{m}$ to $S_{m}$ | - | 0.0833 | $[41]$ |
| $\beta_{mh}$ | Probability of transmission of infection from $I_{m}$ to $S_{h}$ | - | 0.02 | $[41]$ |
| $\theta_{h}$ | Latent period in humans | $/month$ | 0.1 | $[40]$ |
| $\theta_{m}$ | Latent period in mosquitoes | $/month$ | 0.08 | $[40]$ |
| $\delta$ | Disease induced death rate of $I_{h}$ class | $/month$ | 0.0018 | $[40]$ |
| $\psi$ | Disease induced death rate of $I_{h}$ class | $/month$ | 0.0001 | Assumed |
| $\tau_{1}$ | From infectious human class to humans treatment class at health facilities, constant treatment rate of humans | $/month$ | [0, 1] | Estimated from [44,45] |
| $\tau_{2}$ | From infectious human class to humans treatment class with traditional medicines | $/month$ | 0.605 | Assumed |
| $\rho$ | From humans treatment class at health facilities to a susceptible human class, rate of loss of immunity | $/month$ | 0.0166 | [44, 45] |
| $\gamma$ | Recovery rate due to natural immunity and the use of traditional medicines | $/month$ | 0*.*0065 | Assumed |
| $\omega$ | From humans treatment with traditional medicines class to treatment at health facilities, progression rate due to ineffectiveness of traditional medicines | $/month$ | 0.01 | Assumed |
| $\gamma_{1}$ | From a recovered human class to an infectious human class, relapse rate | $/month$ | 0.1 | [44] |
| $\rho_{1}$ | From a recovered human class to a susceptible human class, rate of loss of immunity | $/month$ | 0.0146 | $[41]$ |

# **References**

1. Sato, S. (2021). Plasmodium—a brief introduction to the parasites causing human malaria and their basic biology. *Journal of physiological anthropology*, *40*(1), 1.

2. World Health Organization. (2023). *World malaria report 2023*. World Health Organization.

3. Kwenti, T. E. (2018). Malaria and HIV coinfection in sub-Saharan Africa: prevalence, impact, and treatment strategies. *Research and reports in tropical medicine*, 123-136.

4. Eikenberry, S. E., & Gumel, A. B. (2018). Mathematical modeling of climate change and malaria transmission dynamics: a historical review. *Journal of mathematical biology*, *77*(4), 857-933.

5. Nisar, K. S., Farman, M., Abdel-Aty, M., & Ravichandran, C. (2024). A review of fractional order epidemic models for life sciences problems: Past, present and future. *Alexandria Engineering Journal*, *95*, 283-305.

6. Atangana, A. (2018). Non validity of index law in fractional calculus: a fractional differential operator with Markovian and non-Markovian properties. *Physica A: statistical mechanics and its applications*,*505*,688-706.

7. Owolabi, K. M. (2018). Analysis and numerical simulation of multicomponent system with Atangana–Baleanu fractional derivative. *Chaos, Solitons & Fractals*, *115*, 127-134.

8. Baleanu, D., Shekari, P., Torkzadeh, L., Ranjbar, H., Jajarmi, A., & Nouri, K. (2023). Stability analysis and system properties of Nipah virus transmission: A fractional calculus case study. *Chaos, Solitons & Fractals*, *166*, 112990.

9. Atangana, A., & Baleanu, D. (2016). New fractional derivatives with nonlocal and non-singular kernel: theory and application to heat transfer model. *arXiv preprint arXiv:1602.03408*.

10. Defterli, O., Baleanu, D., Jajarmi, A., Sajjadi, S. S., Alshaikh, N., & Asad, J. H. (2022). Fractional treatment: an accelerated mass-spring system.

11. Gizaw, A. K., & Deressa, C. T. (2024). Analysis of Age‐Structured Mathematical Model of Malaria Transmission Dynamics via Classical and ABC Fractional Operators. *Mathematical Problems in Engineering*, *2024*(1), 3855146.

12. Abidemi, A., & Owolabi, K. M. (2024). Unravelling the dynamics of Lassa fever transmission with nosocomial infections via non-fractional and fractional mathematical models. *The European Physical Journal Plus*, *139*(2), 1-30.

13. Atangana, A., Akgül, A., & Owolabi, K. M. (2020). Analysis of fractal fractional differential equations. *Alexandria Engineering Journal*, *59*(3), 1117-1134.

14. Sado, A. E., & Kotola, B. S. (2024). A mathematical model based on ABC fractional order for TB transmission with treatment interruptions in case of Bule Hora town, Ethiopia. *Informatics in Medicine Unlocked*, *47*, 101498.

15. Tepljakov, A. (2017). *Fractional-order modeling and control of dynamic systems*. Springer.

16. Baleanu, D., Arshad, S., Jajarmi, A., Shokat, W., Ghassabzade, F. A., & Wali, M. (2023). Dynamical behaviours and stability analysis of a generalized fractional model with a real case study. *Journal of Advanced Research*, *48*, 157-173.

17. Owolabi, K. M. (2018). Numerical patterns in reaction–diffusion system with the Caputo and Atangana–Baleanu fractional derivatives. *Chaos, Solitons & Fractals*, *115*, 160-169.

18. Deressa, C. T., & Duressa, G. F. (2021). Analysis of Atangana–Baleanu fractional-order SEAIR epidemic model with optimal control. *Advances in Difference Equations*, *2021*, 1-25.

19. Karaagac, B., Owolabi, K. M., & Nisar, K. S. (2020). Analysis and dynamics of illicit drug use described by fractional derivative with Mittag-Leffler kernel. *CMC-Comput Mater Cont*, *65*(3), 1905-1924.

20. Baleanu, D., Jajarmi, A., Sajjadi, S. S., & Mozyrska, D. (2019). A new fractional model and optimal control of a tumor-immune surveillance with non-singular derivative operator. *Chaos: An Interdisciplinary Journal of Nonlinear Science*, *29*(8).

21. Naik, P. A., Zu, J., & Owolabi, K. M. (2020). Modeling the mechanics of viral kinetics under immune control during primary infection of HIV-1 with treatment in fractional order. *Physica A: statistical mechanics and its applications*, *545*, 123816.

22. Abdoon, M. A., Saadeh, R., Berir, M., & Guma, F. E. (2023). Analysis, modeling and simulation of a fractional-order influenza model. *Alexandria Engineering Journal*, *74*, 231-240.

23. Bhatter, S., Jangid, K., Abidemi, A., Owolabi, K. M., & Purohit, S. D. (2023). A new fractional mathematical model to study the impact of vaccination on COVID-19 outbreaks. *Decision Analytics Journal*, *6*, 100156.

24. Menbiko, D. K., & Deressa, C. T. (2024). Modeling and Analysis of an Age‐Structured Malaria Model in the Sense of Atangana–Baleanu Fractional Operators. *Journal of Mathematics*, *2024*(1), 6652037.

25. Hristov, J. (2019). Response functions in linear viscoelastic constitutive equations and related fractional operators. *Mathematical modelling of natural phenomena*, *14*(3), 305.

26. Vieru, D., Fetecau, C., Ahmed, N., & Shah, N. A. (2021). A generalized kinetic model of the advection-dispersion process in a sorbing medium. *Mathematical Modelling of Natural Phenomena*, *16*, 39.

27. Abro, K. A., & Atangana, A. (2020). A comparative analysis of electromechanical model of piezoelectric actuator through Caputo–Fabrizio and Atangana–Baleanu fractional derivatives. *Mathematical Methods in the Applied Sciences*, *43*(17), 9681-9691.

28. Alzahrani, E., El-Dessoky, M. M., & Baleanu, D. (2021). Mathematical modeling and analysis of the novel Coronavirus using Atangana–Baleanu derivative. *Results in Physics*, *25*, 104240.

29. Yusuf, A., Qureshi, S., & Shah, S. F. (2020). Mathematical analysis for an autonomous financial dynamical system via classical and modern fractional operators. *Chaos, Solitons & Fractals*, *132*, 109552.

30. Atta, D. (2022). Thermal Diffusion Responses in an Infinite Medium with a‎ Spherical Cavity using the Atangana-Baleanu Fractional‎ Operator. *Journal of Applied and Computational Mechanics*, *8*(4),1358-1369.

31. Ochepo, P. A. (2022). Perceptions and attitudes to health-seeking delays for malaria treatment in Makurdi, Nigeria.

32. Thapa, S., & Aro, A. R. (2018). Strategies to integrate community-based traditional and complementary healthcare systems into mainstream HIV prevention programs in resource-limited settings. *Globalization and Health*, *14*, 1-4.

33. Alebie, G., Urga, B., & Worku, A. (2017). Systematic review on traditional medicinal plants used for the treatment of malaria in Ethiopia: trends and perspectives. *Malaria Journal*, *16*, 1-13.

34. Cock, I. E., Selesho, M. I., & Van Vuuren, S. F. (2019). A review of the traditional use of southern African medicinal plants for the treatment of malaria. *Journal of ethnopharmacology*, *245*, 112176.

35. Appiah, E. O., Appiah, S., Oti-Boadi, E., Oppong-Besse, A., Awuah, D. B., Asiedu, P. O., & Oti-Boateng, L. E. (2022). Practices of herbal management of malaria among trading mothers in Shai Osudoku District, Accra. *PloS one*, *17*(7), e0271669.

36. Akilimali, A., Bisimwa, C., Aborode, A. T., Biamba, C., Sironge, L., Balume, A., & Fajemisin, E. A. (2022). Self-medication and Anti-malarial Drug Resistance in the Democratic Republic of the Congo (DRC): A silent threat. *Tropical Medicine and Health*, *50*(1), 73.

37. Willcox, M. L., & Bodeker, G. (2004).Traditional herbal medicines for malaria. *Bmj*, *329*(7475), 1156-1159.

38. Makundi, E. A., Malebo, H. M., Mhame, P., Kitua, A. Y., & Warsame, M. (2006). Role of traditional healers in the management of severe malaria among children below five years of age: the case of Kilosa and Handeni Districts, Tanzania. *Malaria journal*, *5*, 1-9.

39. Olaniyi, S., Falowo, O. D., Okosun, K. O., Mukamuri, M., Obabiyi, O. S., & Adepoju, O. A. (2023). Effect of saturated treatment on malaria spread with optimal intervention. *Alexandria Engineering Journal*, *65*, 443-459.

40. Woldegerima, W. A., Ouifki, R., & Banasiak, J. (2021). Mathematical analysis of the impact of transmission-blocking drugs on the population dynamics of malaria. *Applied Mathematics and Computation*, *400*, 126005.

41. Gebremichael, S. M., & Mekonnen, T. T. (2019). Relapse Effect on the Dynamics of Malaria in Humans and Mosquitoes: A Mathematical Model Analysis. *IOSR Journal of Mathematics*, *15*(5), 46-59.

42. Naik, P. A., Zu, J., & Owolabi, K. M. (2020). Global dynamics of a fractional order model for the transmission of HIV epidemic with optimal control. *Chaos, Solitons & Fractals*, *138*, 109826.

43. Petráš, I. (2011). *Fractional-order nonlinear systems: modeling, analysis and simulation*. Springer Science & Business Media.

44. Mangongo, Y. T., Bukweli, J. D. K., Kampempe, J. D. B., Mabela, R. M., & Munganga, J. M. W. (2022). Stability and global sensitivity analysis of the transmission dynamics of malaria with relapse and ignorant infected humans. *Physica Scripta*, *97*(2), 024002.

45. Aldila, D., & Angelina, M. (2021). Optimal control problem and backward bifurcation on malaria transmission with vector bias. *Heliyon*, *7*(4).

46. Baleanu, D., & Fernandez, A. (2018). On some new properties of fractional derivatives with Mittag-Leffler kernel. *Communications in Nonlinear Science and Numerical Simulation*, *59*, 444-462.

47. Panda, S. K. (2020). Applying fixed point methods and fractional operators in the modelling of novel coronavirus 2019-nCoV/SARS-CoV-2. *Results in Physics*, *19*, 103433.

48. Bakary, T., Boureima, S., & Sado, T. (2018). A mathematical model of malaria transmission in a periodic environment. *Journal of biological dynamics*, *12*(1), 400-432.

49. Brauer, F., Van den Driessche, P., & Allen, L. J. (2008). *Mathematical epidemiology* (Vol. 1945, pp. 3-17). J. Wu (Ed.). Berlin: Springer.

50. Deressa, C. T., Mussa, Y. O., & Duressa, G. F. (2020). Optimal control and sensitivity analysis for transmission dynamics of Coronavirus. Results in Physics, 19, 103642.

51. Chitnis, N., Hyman, J. M., & Cushing, J. M. (2008). Determining important parameters in the spread of malaria through the sensitivity analysis of a mathematical model. *Bulletin of mathematical biology*, *70*, 1272-1296.

52. Toufik, M., & Atangana, A. (2017). New numerical approximation of fractional derivative with non-local and non-singular kernel: application to chaotic models. *The european physical journal plus*, *132*, 1-16.
