# Supplementary material for: A novel ABC fractional-order mathematical model for malaria transmission dynamics incorporating treatment-seeking behavior
Source: PLoS One. 2025 Jun 18;20(6):e0319166. doi: 10.1371/journal.pone.0319166 (PMC12176305; doi:10.1371/journal.pone.0319166)
Supplement: Attachment — Submitted filename: Review reports PONE.pdf [file pone.0319166.s002.pdf]

Review reports PONE-D-24-39540

The use of a fractional-order model is innovative, especially in the context of malaria transmission. Fractional-order models capture memory and hereditary properties, offering a deeper understanding of disease dynamics compared to traditional models. The inclusion of both professional health facilities and indigenous traditional medicine into the model is a significant strength. This dual approach adds realism, as these forms of treatment coexist in many malaria-endemic regions.

The authors provide a thorough mathematical exploration, including the existence and uniqueness of solutions, identification of equilibrium points (malaria-free and malaria-present), and detailed numerical simulations. This offers a solid theoretical foundation for the model. The study addresses a crucial public health issue, emphasizing the need for collaboration between traditional and professional healthcare practices, which is a relevant recommendation for policy-making in malaria-endemic regions. The analysis reveals that fractional-order effects play a crucial role in the progression of the disease. This finding is significant and opens up new avenues for understanding how the disease evolves under different treatment and intervention strategies.

Comments (minor/major) for improvement

1. While the study is mathematically rigorous, the narrative flow could benefit from better structure and clarity, particularly for readers unfamiliar with fractional calculus. A more accessible introduction to fractional-order models and their relevance in epidemiological studies might help.
2. What informs your choice of using the ABC operator in modeling the transmission of malaria? Highlight some of the useful applications of the ABC derivatives to modeling of real-life scenarios.
3. Reference materials on the ABC and other fractional operatorsis inadequate, addition of the following materials will boost the readership of your work: https://doi.org/10.1140/epjp/s13360-024-04910-z; https://doi.org/10.32604/cmc.2020.011623 https://doi.org/10.1016/j.dajour.2022.100156; https://doi.org/10.1016/j.chaos.2020.109826; https://doi.org/10.1016/j.aej.2020.01.005; https://doi.org/10.1016/j.physa.2019.123816; https://doi.org/10.1016/j.chaos.2018.08.022; https://doi.org/10.1016/j.chaos.2018.07.034; https://doi.org/10.1016/j.chaos.2018.08.025
4. The numerical simulations section could be expanded with more detailed explanations of the algorithms or methods used. For example, specifying which numerical schemes were employed for solving the fractional-order system would enhance reproducibility.
5. While the model includes various parameters, a formal sensitivity analysis would be beneficial. Understanding which parameters (such as the rate of treatment or transmission rates) most significantly affect disease dynamics would provide valuable insights into which interventions are most effective.
6. The role of indigenous traditional medicine is mentioned, but the discussion on how it affects long-term disease management could be expanded. Are there any specific case studies or data

that support the claims about temporary recovery and relapse? This would add depth to the discussion on traditional medicine's role in malaria treatment.

7. The study would be stronger if it included a comparison of the model’s predictions with actual data from malaria-endemic regions. Incorporating data-driven validation would further substantiate the conclusions drawn from the model.

8. The paper provides a valuable discussion on public health initiatives, but more concrete policy recommendations based on the model’s findings could be included. This would help in translating the mathematical results into actionable health strategies.

Minor concerns

- It would be useful to include a clearer or visualizations of the model's numerical simulations. There are some cases where tiny lines were used.

- It is obvious that solution below $\alpha=0.8$ appears invisible, explain what makes solution in the range [0.1, 0.8] impracticable. This would help to better illustrate how the fractional-order dynamics impact disease progression over time.

- Ensure that all variables and parameters are consistently defined, and consider simplifying the notation where possible to avoid overwhelming the reader.

Finally, this paper presents a novel and important contribution to the study of malaria transmission dynamics using the concept of fractional calculus. The model’s incorporation of treatment-seeking behavior and traditional medicine interventions reflects real-world complexities and offers valuable insights for public health strategies. With clearer exposition, sensitivity analysis, and validation with empirical data, the manuscript has the potential to make a significant impact in the field of epidemiological modeling. I would recommend a revision for this work.
