# Supplementary material for: A novel ABC fractional-order mathematical model for malaria transmission dynamics incorporating treatment-seeking behavior
Source: PLoS One. 2025 Jun 18;20(6):e0319166. doi: 10.1371/journal.pone.0319166 (PMC12176305; doi:10.1371/journal.pone.0319166)
Supplement: Attachment — Submitted filename: PONE-D-24-39540.pdf [file pone.0319166.s003.pdf]

In this paper, the authors have introduced an advanced mathematical model to analyse malaria transmission. It focuses on integrating the Atangana-Baleanu Caputo (ABC) fractional-order derivative into a malaria model. The contents of the paper are good and contain new ideas. Anyhow, I would like to see the following modifications, whether minor or major, in the revised version, which would increase the strength of the paper and increase its potential readers, as well as improve the current work.

1. The abstract is dense and could benefit from more clarity, especially in explaining the novelty and significance of the approach. Simplifying the language and explicitly stating the problem, solution, and main contributions could make it more accessible.
2. The introduction is comprehensive but might overwhelm the reader with too much background information. Consider condensing the literature review and focusing more on the specific gaps this paper addresses. The introduction should end with a clear statement of the research objectives and contributions.
3. The transitions between sections are abrupt.
4. Include more detailed information about the software or programming languages used for simulations and ensure that all mathematical techniques can be replicated by other researchers.
5. Add a section on limitations, such as potential biases in the data, challenges in modelling human behaviour, or the applicability of the model to other diseases.
6. Strengthen citation practices by referencing foundational work where applicable, particularly for sections that discuss mathematical techniques and the role of traditional medicine in malaria treatment.
7. Include a more thorough discussion of how the model’s findings can inform public health strategies, particularly for integrating traditional and modern medicine.

These revisions should help in clarifying the paper's contributions, improving its readability, and strengthening the technical arguments
