# Supplementary material for: A novel ABC fractional-order mathematical model for malaria transmission dynamics incorporating treatment-seeking behavior
Source: PLoS One. 2025 Jun 18;20(6):e0319166. doi: 10.1371/journal.pone.0319166 (PMC12176305; doi:10.1371/journal.pone.0319166)
Supplement: Attachment — Submitted filename: Response to_Reviewer #3.docx [file pone.0319166.s005.docx]

***Subject: Rebuttal Letter for revised manuscript***

**Re: Manuscript ID [PONE-D-24-39540] - "A Novel ABC Fractional-Order Mathematical Model for Malaria Transmission Dynamics Incorporating Treatment-Seeking Behavior"**

Dear Behzad Ghanbari and Reviewer #3,

We sincerely thank you for the thoughtful and constructive feedback on our manuscript. We have carefully addressed the concerns raised and believe that the revisions enhance the quality, clarity, and impact of our study. Below, we provide a detailed point-by-point response to the comments, highlighting the revisions made in the manuscript.

***Reviewer #3 Comments and our responses***

In this paper, the authors have introduced an advanced mathematical model to analyse malaria transmission. It focuses on integrating the Atangana-Baleanu Caputo (ABC) fractional-order derivative into a malaria model. The contents of the paper are good and contain new ideas. Anyhow, I would like to see the following modifications, whether minor or major, in the revised version, which would increase the strength of the paper and increase its potential readers, as well as improve the current work.

**1.** The abstract is dense and could benefit from more clarity, especially in explaining the novelty and significance of the approach. Simplifying the language and explicitly stating the problem, solution, and main contributions could make it more accessible.

***Response:* Thank you for your feedback, and w**e have rewritten the abstract to simplify the language and enhance clarity, explicitly highlighting the problem, solution, and main contributions. The revised abstract now focuses on the novelty and significance of incorporating the ABC fractional-order derivative in our malaria transmission model and its implications for public health strategies.

**2.** The introduction is comprehensive but might overwhelm the reader with too much background information. Consider condensing the literature review and focusing more on the specific gaps this paper addresses. The introduction should end with a clear statement of the research objectives and contributions.

***Response:* Thank you for your feedback, and** the introduction has been condensed [Section 1 of the revised manuscript] to streamline the background information. We have emphasized the specific research gaps and concluded the section with a concise statement of our objectives and contributions, as per your suggestion.

**3.** The transitions between sections are abrupt.

***Response:*** Thank you for this suggestion. We have carefully revised the manuscript to include logical linking sentences between sections, ensuring smoother transitions and better readability.

**4.** Include more detailed information about the software or programming languages used for simulations and ensure that all mathematical techniques can be replicated by other researchers.

***Response:*** Thank you for this suggestion. We have added a detailed description of the numerical techniques and the software used for simulations in the revised manuscript section [Section 6]. Specifically, the revised manuscript mentions that MATLAB R2023a was utilized for numerical simulations and provides initial population values and parameter settings to facilitate replication by other researchers.

**5.** Add a section on limitations, such as potential biases in the data, challenges in modelling human behaviour, or the applicability of the model to other diseases.

***Response:*** We appreciate your suggestion. We have added a section discussing the study's limitations in the revised manuscript of section [Section 9]. The limitations section addresses potential biases in secondary data, challenges in modeling human behavior, and the applicability of our model to other diseases.

**6.** Strengthen citation practices by referencing foundational work where applicable, particularly for sections that discuss mathematical techniques and the role of traditional medicine in malaria treatment.

***Response:* Thank you for your feedback.** We have included additional citations to foundational work in both mathematical modeling techniques and the discussion of traditional medicine's role in malaria treatment.

**7.** Include a more thorough discussion of how the model’s findings can inform public health strategies, particularly for integrating traditional and modern medicine.

***Response:*** Thank you for this suggestion. The discussion section has been expanded in the revised manuscript [Section 7, page 29] to elaborate on the implications of our findings for public health strategies. We emphasize the importance of integrating traditional and modern medicine to ensure effective and safe malaria treatment, highlighting potential policy recommendations and collaborative approaches.

We hope these revisions address all the concerns raised by the reviewer. We sincerely thank the reviewer and the editorial team for their valuable feedback, which has significantly strengthened our manuscript. The manuscript is now better aligned with the goals of *PLOS ONE*, providing insights into both the theoretical and practical aspects of malaria transmission dynamics.

***Thank you for considering our revised submission. We hope the changes meet the expectations of the reviewers and the editorial board. Please let us know if there are any additional comments or suggestions.***

*Sincerely,*

*Sisay Fikadu Jaleta(Corresponding author)*

*Debre Berhan University,Debre Berhan, Ethiopia*

*Email:* [*sisayfikadu28@gmail.com*](mailto:sisayfikadu28@gmail.com)

*Gemechis File Duressa (Co-author)*

*Department of Mathematics, Jimma University, Jimma, Ethiopia*

*Email:* [*gammeef@gmail.com*](mailto:gammeef@gmail.com)

*Chernet Tuge Deressa (Co-author)*

*Department of Mathematics, Jimma University, Jimma, Ethiopia*

*Email:* [*chernet.deressa@ju.edu.et*](mailto:chernet.deressa@ju.edu.et)
